# Chemoradiotherapy versus radiotherapy in high risk salivary gland cancer

**DOI:** 10.1186/s12957-024-03456-9

**Published:** 2024-07-11

**Authors:** Yicheng Shen, Jiebo Shan

**Affiliations:** Department of Stomatology, Affiliated Hospital of Shaoxing University, Shaoxing, 312000 China

**Keywords:** Salivary gland cancer, Chemoradiotherapy, Radiotherapy, Lymph node metastasis burden, Survival

## Abstract

**Objective:**

The aim of this study was to investigate the potential survival benefits associated with chemoradiotherapy (CRT) as opposed to radiotherapy (RT) in patients with resected high-risk salivary gland cancer (SGC), with a specific focus on determining whether these benefits are influenced by the number of high-risk variables.

**Methods:**

Patients who underwent surgical treatment for high-risk SGC were retrospectively enrolled and categorized into either CRT or RT groups. The impact of adjuvant therapy on locoregional control (LRC) and overall survival (OS) was assessed using a multivariable Cox model.

**Results:**

A total of 152 patients were included following propensity score-matching. In comparison to RT, CRT did not demonstrate a significant survival advantage in terms of LRC (*p* = 0.485, HR: 1.14, 95%CI: 0.36–4.22) and OS (*p* = 0.367, HR: 0.99, 95%CI: 0.17–3.87) in entire population. But among patients with T3/4 stage, high-grade tumors, and 5 or more positive lymph nodes, the addition of chemotherapy to RT significantly (*p* = 0.042) correlated with a 15% reduction in the risk of cancer recurrence (95%CI: 4-54%). Conversely, in other subgroups with varying combinations of high-risk variables, CRT did not provide additional survival benefits for LRC and OS compared to RT.

**Conclusion:**

Adjuvant chemotherapy may be considered in conjunction with RT specifically in cases where there is a presence of T3/4 stage, high-grade tumors, and 5 or more metastatic lymph nodes in high-risk SGC.

**Supplementary Information:**

The online version contains supplementary material available at 10.1186/s12957-024-03456-9.

## Introduction

Salivary gland cancer (SGC) is relatively rare, comprising only about 5% of all malignancies in the head and neck region [1], but it consists of 24 pathologic types based on the newest WHO classification [2]. Surgery accounts for the mainstay of treatment, according to the NCCN guideline, adjuvant radiotherapy (RT) is usually recommended if there are adverse pathologic features [3], extensive literature has confirmed RT contributes to improved locoregional control (LRC), disease specific survival, and overall survival (OS) in cases presenting stage T3/4, lymph node (LN) metastasis, high pathologic grade, positive margin, perineural invasion (PNI), lymphovascular invasion (LVI), and extranodal extension (ENE) [4–8].

Current evidence regarding adjuvant chemoradiotherapy (CRT) for high-risk SGCs is notably scarce, being classified only as category 2B evidence in NCCN guideline [3]. Clinicians resorting to adjuvant CRT for SGCs often draw upon insights derived from two randomized clinical trials and subsequent pooled analyses that have demonstrated improved outcomes with postoperative CRT compared to RT alone in cases of head and neck squamous cell carcinoma [9–11]. Although it’s crucial to recognize that these pivotal trials have specifically excluded malignant neoplasms of the salivary gland, ENE and positive margin have been considered as factors warranting the addition of chemotherapy to RT in head and neck cancer. Nonetheless, the applicability of these findings to SGCs remains uncertain due to the absence of high quality evidence.

The issue of survival benefit associated with CRT in SGCs is a subject of debate within the scientific community. While some researchers have failed to establish a clear association between chemotherapy and prognosis [7, 12–14], others have observed that, compared to RT alone, CRT may indeed lead to improved outcomes in certain specific pathologic types [8, 15]. This discrepancy underscores the complexity of treating SGCs and highlights the need for further research to elucidate the role of adjuvant CRT in optimizing therapeutic outcomes for this rare malignancy.

Therefore, our aim was to scrutinize the survival advantage linked with CRT compared to RT alone in high-risk SGC, and specifically to discern whether this benefit is influenced by the burden of LN metastasis and the number of high-risk variables.

## Patients and methods

### Ethical approval

This study was approved by Affiliated Hospital of Shaoxing University Institutional Research Committee, and written informed consent for medical research was obtained from all patients before starting the treatment. All methods were performed in accordance with the relevant guidelines and regulations.

### Study design

**Fig. 1:**
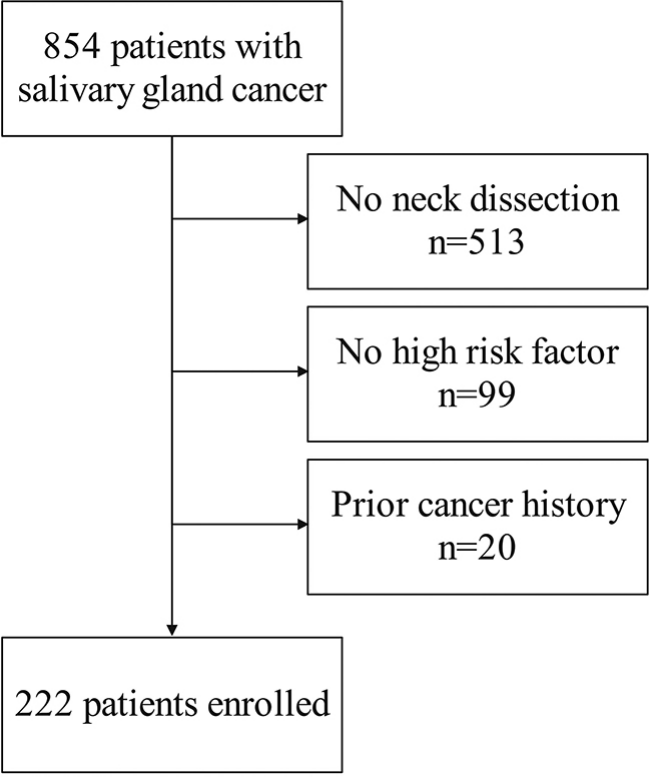
Flowchart of the enrolled patients

To fulfill our research objective, a retrospective analysis according to the STROBE Checklist for case-control studies (https://www.strobe-statement.org/checklists/) was conducted on the medical records of individuals who underwent surgical treatment for primary SGC from January 2000 to December 2023. Inclusion criteria were defined as follows: all patients must have undergone neck dissection, exhibited at least one high-risk factor, and had available follow-up data. Exclusion criteria involved individuals with a prior history of malignancy (Fig. 1). A comprehensive dataset encompassing demographic details, pathological characteristics, treatment modalities employed, and subsequent follow-up information for the enrolled patients was documented.

### Variable definition

**Fig. 2:**
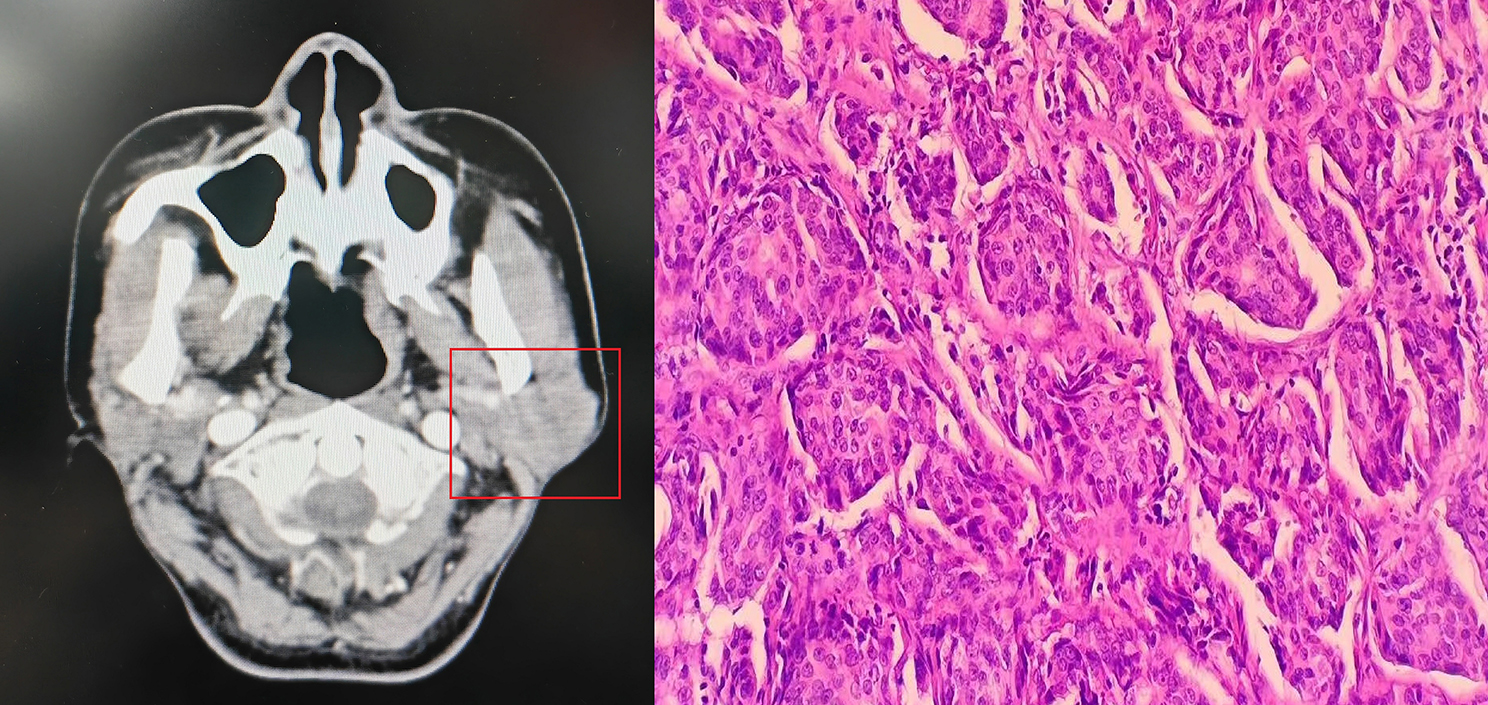
Radiologic and pathologic images of one patient with salivary duct carcinoma

Every pathologic specimen underwent review by a panel of at least two proficient head and neck pathologists to ensure accurate diagnosis. High-risk factors encompassed in this study were delineated as follows: pT3/4 classification, presence of LN metastasis, high histologic grade (Fig. 2), PNI, LVI, ENE, and positive margin, consistent with established literature [7, 8, 10]. Tumor staging was determined utilizing the 8th edition of the AJCC classification, and pathological grade was stratified into low, intermediate, and high categories [2]. LVI was designated when tumor cells were identified within the lymphatic vessels, while confirmation of PNI required evidence of tumor cell infiltration into nerve tissue. ENE diagnosis was established upon the detection of cancerous cells extending beyond the boundaries of the LN capsule. Margin was positive if there were cancerous cells present at the outer edge or margin of the removed tissue specimen.

**Fig. 3:**
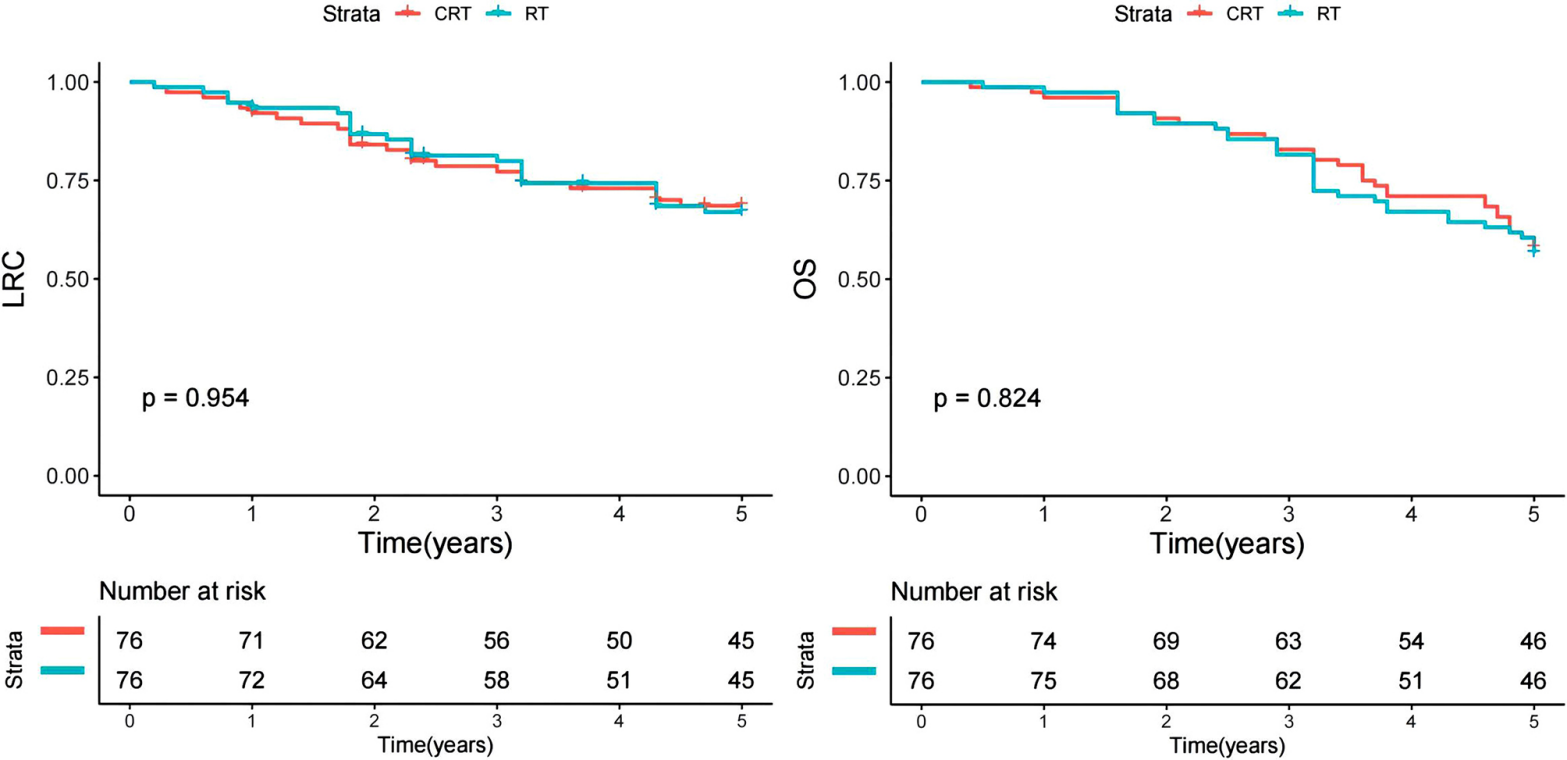
Kaplan-Meier analysis of locoregional control (LRC) and overall survival (OS) between chemoradiotherapy (CRT) and radiotherapy (RT) groups

Primary outcome variables were the 5-year LRC and OS rates. Time to locoregional control was calculated from the date of surgery to the occurrence of the first locoregional recurrence or the most recent follow-up appointment. Meanwhile, time to overall survival was computed from the date of surgery to the documented date of death or the latest follow-up encounter.

### Treatment principle

Each patient underwent a thorough evaluation comprising ultrasound and CT scans as a baseline (Fig. 2), with occasional utilization of PET-CT scans for assessment of both cervical and distant metastases. Frozen sections of the primary tumor and margins were consistently analyzed during surgical procedures to ensure precise pathological evaluation. Neck dissection was performed whenever there were clinically or pathologically identified positive LNs. For non-parotid gland cancers, the extent of neck dissection ranged from levels I to III, while for parotid gland cancers, levels I to III and Va were routinely included in the dissection field. This comprehensive approach aimed to achieve optimal oncologic outcomes and minimize the risk of locoregional recurrence. In cases where high-risk factors were identified, adjuvant RT was recommended as part of the treatment regimen, the clinical target volume included the tumor bed with a 1–2 cm margin, and a dose of 60–66 Gy was prescribed. The presence of ENE or positive margins warranted the consideration of adjuvant chemotherapy (platinum or paclitaxel based, administered over 4–6 cycles) to escalate the therapeutic intensity and enhance treatment efficacy.

### Sample size calculation

In order to ascertain the minimum sample size necessary to detect potential differences in survival outcomes, the 5-year OS rates were estimated at 45% and 75% for the RT and CRT groups, respectively, drawing from previous literature sources [4, 5]. Utilizing a two-sided α value of 0.05 and a β value of 0.9, it was determined that a minimum of approximately 60 patients would be essential for each group to achieve statistical power.

### Statistic analysis

The absence of data within the variables of tumor stage, pathologic grade, PNI, and LVI exhibited a non-random missing pattern [16]. The rates of missing data varied with percentages recorded at 13.2% for pathologic grade, 10.4% for tumor stage, 9.0% for PNI, and 8.2% for LVI. To address these missing data points, a rigorous imputation process was undertaken utilizing the Multiple Imputation by Chained Equations algorithm with Fully Conditional Specifications [17].

Patients were categorized into two groups based on the type of adjuvant treatment administered: RT or CRT. Disparities in clinicopathologic variables between these groups were scrutinized utilizing the Chi-square test to discern significant differences. To mitigate potential biases inherent in observational studies, a propensity score-matching (PSM) technique was deployed to establish well-matched cohorts, with matching executed in a 1:1 ratio via the nearest neighbor approach.

The impact of clinicopathologic variables on prognosis was evaluated through both univariate and multivariable Cox regression models, elucidating hazard ratios (HR) alongside their corresponding 95% confidence intervals (CI). Subsequently, the prognostic significance of these variables was further explored by stratifying patients based on the number of metastatic LNs and the presence of high-risk factors. Survival rates were computed utilizing the Kaplan-Meier method. All statistical analyses were conducted using R version 3.4.4, with statistical significance established at a threshold of a p-value below 0.05.

## Results

### Baseline date

A total of 222 patients were included in the study, with a mean age of 50 ± 14 years. Among them, 92 (41.4%) were male and 130 (58.6%) were female. The preponderance of cases (*n* = 178, 80.2%) arose in the major salivary gland. Pathologic tumor staging revealed T1/2 in 100 (45.0%) patients and T3/4 in 122 (55.0%) patients. The most common histologic type was mucoepidermoid carcinoma (*n* = 118, 53.2%), and the least frequent type was epithelial-myoepithelial carcinoma (*n* = 2, 1.8%) (Supplementary Table 1). Tumor grades were classified as low, intermediate, and high in 45 (20.3%), 102 (45.9%), and 75 (33.8%) patients, respectively. PNI and LVI were observed in 57 (25.7%) and 45 (20.3%) patients, respectively. Pathologic neck staging indicated N0 in 25 (11.3%) patients, N1 in 110 (49.5%) patients, and N2/3 in 87 (39.2%) patients, with involvement of level IV/V present in 51 (23.0%) patients. ENE was present in 66 (29.7%) cases, while positive margins were noted in 25 (11.3%) patients. Additionally, 65 (29.3%) patients exhibited more than 4 metastatic LNs.

The CRT cohort comprised 76 patients, demonstrating a higher prevalence of N2/3 neck stages (*p* < 0.001), a greater incidence of ENE (*p* = 0.009), and a heightened burden of LN metastasis (*p* = 0.016) compared to the RT group. Nonetheless, both groups exhibited similar distributions with respect to other variables (all *p* > 0.05, Table 1). Subsequently, the variables of neck staging, ENE presence, and the number of metastatic LNs were evaluated through propensity score-matching, resulting in a total of 152 patients (76 in each group) for further analysis of survival outcomes (Supplementary Table 2).

**Table 1:** Baseline data of enrolled patients treated by radiotherapy (RT) or chemoradiotherapy (CRT) after resection

Variable	RT (*n* = 146)	CRT (*n* = 76)	*p*
Age			
≤50	79 (54.1%)	38 (50.0%)	
>50	67 (45.9%)	38 (50.0%)	0.561
Sex			
Male	64 (43.8%)	28 (36.8%)	
Female	82 (56.2%)	48 (63.2%)	0.316
Primary site			
Major	114 (78.1%)	64 (84.2%)	
Minor	32 (21.9%)	12 (15.8%)	0.277
Tumor stage			
T1 + T2	65 (44.5%)	35 (46.0%)	
T3 + T4	81 (55.5%)	41 (54.0%)	0.828
Perineural invasion			
No	111 (76.0%)	54 (71.0%)	
Yes	35 (24.0%)	22 (29.0%)	0.421
Lymphovascular invasion			
No	119 (81.5%)	58 (76.3%)	
Yes	27 (19.5%)	18 (23.7%)	0.361
Histologic grade			
Low	33 (22.6%)	12 (15.8%)	
Intermediate	67 (45.9%)	35 (46.1%)	
High	46 (31.5%)	29 (38.2%)	0.407
Neck stage			
N0	25 (17.1%)	0	
N1	73 (50.0%)	37 (48.7%)	
N2/3	48 (32.9%)	39 (51.3%)	< 0.001
Level IV/V involvement			
No	117 (80.1%)	54 (71.1%)	
Yes	29 (19.9%)	22 (28.9%)	0.127
Extranodal extension			
No	111 (76.0%)	45 (59.2%)	
Yes	35 (24.0%)	31 (40.8%)	0.009
Margin			
Negative	130 (89.0%)	67 (88.2%)	
Positive	16 (11.0%)	9 (11.8%)	0.843
Number of metastatic lymph nodes			
≤4	111 (76.0%)	46 (60.5%)	
>4	35 (24.0%)	30 (39.5%)	0.016

Following a median follow-up period of 5 years (range: 0.2–10), there were documented 47 instances of locoregional recurrences and 65 deaths. The 5-year LRC rates stood at 68% (95%CI: 58-78%) for the CRT group and 67% (95%CI: 55-79%) for the RT group. Notably, the observed difference between the two groups was not statistically significant (Fig. 3, *p* = 0.954). Similarly, the 5-year OS rates were recorded as 57% (95%CI: 45-69%) for the CRT group and 54% (95%CI: 42-66%) for the RT group. Once again, the noted disparity between the groups did not reach statistical significance (Fig. 3, *p* = 0.824).

### Univariate analysis

When compared to RT, CRT did not demonstrate a discernible survival advantage in terms of LRC (*p* = 0.485, HR: 1.14, 95%CI: 0.36–4.22) and OS (*p* = 0.367, HR: 0.99, 95%CI: 0.17–3.87). T3/4 stage, LVI, high pathologic grade, N2/3 stage, positive margins, and the presence of 5 or more metastatic LNs were notably predictive of poorer LRC and OS outcomes, while other variables exhibited limited impact on survival results (Table 2). Subsequently, these six factors were subjected to further evaluation in a multivariable Cox model.

**Table 2:** Univariate Cox analysis of predictors for locoregional control (LRC) and overall survival (OS)

Variable	LRC		OS	
	*P*	HR [95%CI]	*P*	HR [95%CI]
Age				
≤50		ref		ref
>50	0.547	2.16 [0.73–5.90]	0.356	2.00 [0.62–4.65]
Sex				
Male				
Female	0.222	1.23 [0.47–7.12]	0.317	1.87 [0.41–8.10]
Primary site				
Major		ref		ref
Minor	0.425	2.37 [0.83–7.73]	0.321	2.64 [0.77–8.11]
Tumor stage				
T1 + T2		ref		ref
T3 + T4	0.005	3.10 [1.22–9.05]	0.007	2.90 [1.17–7.36]
Perineural invasion				
No		ref		ref
Yes	0.126	2.45 [0.63–10.08]	0.243	2.76 [0.86–9.99]
Lymphovascular invasion				
No		ref		ref
Yes	0.014	2.72 [1.35–8.17]	0.015	3.00 [1.74–9.45]
Histologic grade				
Low		ref		ref
Intermediate	0.178	1.98 [0.32–11.67]	0.435	2.27 [0.46–17.22]
High	< 0.001	3.86 [1.88–17.13]	< 0.001	4.07 [1.65–20.67]
Neck stage				
N1		ref		ref
N2/3	0.017	2.17 [1.65–6.43]	0.026	2.33 [1.70–7.57]
Level IV/V involvement				
No		ref		ref
Yes	0.156	2.28 [0.65–8.10]	0.474	3.08 [0.46–7.76]
Extranodal extension				
No		ref		ref
Yes	0.532	1.89 [0.66–14.27]	0.482	2.00 [0.59–16.10]
Margin				
Negative		ref		ref
Positive	< 0.001	4.27 [2.21–20.99]	< 0.001	5.03 [1.86–44.62]
Number of metastatic LNs*				
≤4		ref		ref
>4	< 0.001	3.19 [1.64–15.23]	< 0.001	3.48 [1.95–18.25]
Adjuvant therapy				
Radiotherapy		ref		ref
Chemoradiotherapy	0.485	1.14 [0.36–4.22]	0.367	0.99 [0.17–3.87]

* LN: lymph node

### Multivariable analysis (Table 3)

**Table 3:** Multivariable Cox analysis of predictors for locoregional control (LRC) and overall survival (OS)

Variable	LRC		OS	
	*P*	HR [95%CI]	*P*	HR [95%CI]
Tumor stage				
T1 + T2		ref		ref
T3 + T4	0.015	2.19 [1.54–6.28]	0.010	2.25 [1.67–7.18]
Lymphovascular invasion				
No		ref		ref
Yes	0.342	1.78 [0.72–5.37]	0.224	2.04 [0.83–8.34]
Histologic grade				
Low		ref		ref
Intermediate	0.167	1.65 [0.71–5.26]	0.209	1.90 [0.66–7.05]
High	< 0.001	3.27 [1.32–8.11]	< 0.001	3.08 [1.41–9.17]
Neck stage				
N1		ref		ref
N2/3	0.163	1.99 [0.72–5.64]	0.218	2.15 [0.72–8.84]
Margin				
Negative		ref		ref
Positive	< 0.001	4.27 [1.90−12.34]	< 0.001	5.43 [2.21–17.15]
Number of metastatic LNs*				
≤4		ref		ref
>4	0.007	2.19 [1.52–6.29]	0.011	2.35 [1.36–7.36]

* LN: lymph node

In the Cox model analysis for LRC, the HR was 2.19 (95%CI: 1.54–6.28) for T3/4 stage, indicating a significantly higher risk (*p* = 0.015) compared to the T1/2 group. While intermediate grade tumors exhibited a similar impact on LRC (*p* = 0.167, HR: 1.65, 95%CI: 0.71–5.26) as low grade tumors, high grade malignancies conferred a 2.2-fold increased risk of recurrence. Presence of positive margins was linked with approximately a 4-fold higher likelihood of disease recurrence compared to negative margins. Patients with more than 4 metastatic LNs had a HR of 2.19 (95%CI: 1.52–6.29) for recurrence risk relative to those with ≤ 4 positive LNs, with this difference proving to be statistically significant (*p* = 0.007). Either LVI or N2/3 stage did not retain their independent impact on LRC outcomes.

In the analysis for OS using the Cox model, the HR was 2.25 (95%CI: 1.67–7.18) for T3/4 stage, significantly higher (*p* = 0.010) than that of the T1/2 group. While intermediate grade tumors exhibited a comparable effect on OS (*p* = 0.209, HR: 1.90, 95%CI: 0.66–7.05) to low grade tumors, high grade malignancies were associated with a 2-fold increased risk of mortality. Positive margins were linked with approximately a 4-fold higher probability of death compared to negative margins. Patients with more than 4 metastatic LNs had a HR of 2.35 (95%CI: 1.36–7.36) for mortality risk compared to those with ≤ 4 positive LNs, with this difference also being statistically significant (*p* = 0.011). Notably, either LVI or N2/3 stage did not exhibit independent effects on OS.

### Subgroup analysis

In order to assess whether the comparison between CRT and RT outcomes varied based on the presence of multiple high-risk factors, a comprehensive subgroup analysis was conducted. Specifically focusing on LRC outcomes (Table 4), it was observed that among patients characterized by T3/4 stage, high-grade tumors, and 5 or more positive lymph nodes, the incorporation of chemotherapy alongside RT was significantly (*p* = 0.042) linked to a 15% reduction in the risk of cancer recurrence (95%CI: 4-54%). However, in other subgroups presenting different combinations of high-risk variables, CRT did not confer added survival benefits over RT (all *p* > 0.05).

**Table 4:** Subgroup analysis of impact of adjuvant therapy on locoregional control stratified by number of high-risk factors and metastatic lymph nodes (LNs)

Variable	*p*	HR [95%CI]
**One high-risk factor**		
T3/4		
RT*		ref
CRT	0.673	1.22 [0.54–5.89]
High grade		
RT		ref
CRT	0.723	2.56 [0.36–9.03]
Positive margin		
RT		ref
CRT	0.665	1.86 [0.45–19.05]
Number of metastatic LNs > 4^&^		
RT		ref
CRT	0.234	1.67 [0.48–5.11]
**Two high-risk factors**		
Positive margin + T3/4		
RT		ref
CRT	0.648	2.11 [0.35–7.62]
Positive margin + high grade		
RT		ref
CRT	0.821	1.05 [0.61–7.09]
Positive margin + number of metastatic LNs > 4		
RT		ref
CRT	0.775	1.85 [0.77–6.25]
T3/4 + high grade		
RT		ref
CRT	0.176	1.35 [0.62–9.32]
T3/4 + number of metastatic LNs > 4		
RT		ref
CRT	0.375	1.27 [0.45–7.28]
High grade + number of metastatic LNs > 4		
RT		ref
CRT	0.135	1.82 [0.41–6.96]
**Three high-risk factors**		
Positive margin + T3/4 + high grade		
RT		ref
CRT	0.784	2.00 [0.19–8.18]
Positive margin + T3/4 + number of metastatic LNs > 4		
RT		ref
CRT	0.745	1.98 [0.23–9.32]
Positive margin + high grade + number of metastatic LNs > 4		
RT		ref
CRT	0.935	1.07 [0.35–6.27]
T3/4 + high grade + number of metastatic LNs > 4		
RT		ref
CRT	0.042	0.85 [0.46–0.96]
**Four high-risk factors**		
RT		ref
CRT	0.876	1.78 [0.13–9.18]

* RT: radiotherapy; CRT: chemoradiotherapy;

& LN: lymph node

In the evaluation of OS, the supplementary administration of chemotherapy alongside RT did not result in a significant alteration in OS compared to RT alone, regardless of the high-risk factors present among patients (all *p* > 0.05, Table 5).

**Table 5:** Subgroup analysis of impact of adjuvant therapy on overall survival stratified by number of high-risk factors and metastatic lymph nodes (LNs)

Variable	*p*	HR [95%CI]
**One high-risk factor**		
T3/4		
RT*		ref
CRT	0.564	1.64 [0.46–6.83]
High grade		
RT		ref
CRT	0.663	2.24 [0.43–8.17]
Positive margin		
RT		ref
CRT	0.509	1.67 [0.53–12.13]
Number of metastatic LNs > 4^&^		
RT		ref
CRT	0.356	1.82 [0.32–6.27]
**Two high-risk factors**		
Positive margin + T3/4		
RT		ref
CRT	0.564	2.09 [0.33–6.98]
Positive margin + high grade		
RT		ref
CRT	0.761	1.21 [0.48–6.33]
Positive margin + number of metastatic LNs > 4		
RT		ref
CRT	0.615	1.43 [0.66–7.81]
T3/4 + high grade		
RT		ref
CRT	0.223	1.25 [0.47–7.67]
T3/4 + number of metastatic LNs > 4		
RT		ref
CRT	0.434	1.34 [0.37–6.56]
High grade + number of metastatic LNs > 4		
RT		ref
CRT	0.209	1.76 [0.36–7.09]
**Three high-risk factors**		
Positive margin + T3/4 + high grade		
RT		ref
CRT	0.654	1.98 [0.45–10.23]
Positive margin + T3/4 + number of metastatic LNs > 4		
RT		ref
CRT	0.356	1.69 [0.45–8.67]
Positive margin + high grade + number of metastatic LNs > 4		
RT		ref
CRT	0.674	1.23 [0.43–7.45]
T3/4 + high grade + number of metastatic LNs > 4		
RT		ref
CRT	0.134	0.88 [0.46–5.78]
**Four high-risk factors**		
RT		ref
CRT	0.683	1.89 [0.32–7.24]

* RT: radiotherapy; CRT: chemoradiotherapy;

& LN: lymph node

## Discussion

Our key findings revealed that, within the entire sample, CRT did not exhibit a notably superior outcome in terms of LRC or OS compared to RT. However, in patients diagnosed with T3/4 stage, high-grade tumors, and 5 or more positive lymph nodes, the integration of chemotherapy with RT correlated with enhanced LRC. This study has thus shed light on the ideal candidates for CRT, thereby influencing clinical decisions regarding the management of high-risk SGC.

Chemotherapy plays a crucial role as an adjunct in the treatment of head and neck cancer, reducing the risk of locoregional and distant metastasis while enhancing radiation sensitivity. The current guidelines for CRT are primarily based on the findings of two pivotal clinical trials and their subsequent combined analyses [9–11]. The EORTC 22,931 trial [9] involved the random allocation of 167 patients with resected stage III/IV head and neck cancer into either RT or CRT groups. The subsequent survival analysis revealed that the addition of chemotherapy to RT was associated with superior 5-year rates of progression-free survival, LRC, and OS, with similar mucosal adverse effects observed in both groups. Similarly, the RTOG trial [10] also divided 459 patients into comparable groups, demonstrating a 39% reduction in the risk of locoregional recurrence with the addition of chemotherapy in the CRT arm, along with significant improvements in disease-free survival. A combined analysis conducted by Bernier et al. [11] further validated the identification of ENE and positive margins as critical prognostic factors for unfavorable outcomes. While these investigations did not specifically address SGCs, ENE and positive margins have since become widely recognized as key determinants for the incorporation of CRT in the management of head and neck cancer. Nevertheless, ongoing debates persist due to the inherent chemoresistance often associated with SGCs, as evidenced by objective response rates ranging from 0 to 44% [18].

The comparison between CRT and RT in terms of prognosis for SGC has been extensively investigated. Amini et al. [12] conducted a comprehensive analysis of 2210 SGC patients sourced from the National Cancer Database (NCDB), with 1842 patients receiving RT and the remainder undergoing CRT. Interestingly, upon multivariable analysis, it was revealed that OS was notably compromised with adjuvant CRT (HR: 1.22, 95%CI: 1.03–1.44) compared to RT alone, a trend consistent even after propensity score matching (HR: 1.20, 95%CI: 0.98–1.47). In a study by Cheraghlou et al. [4], a cohort of 8580 SGC patients from the same database was stratified into four groups based on cancer stage (early vs. late) and the presence of adverse features. Among patients with early-stage disease and adverse features, a treatment regimen involving surgery and adjuvant therapy was linked to enhanced OS compared to surgery alone. However, due to limited sample sizes, the authors did not directly contrast the outcomes of RT versus CRT. Notably, in cases of late-stage disease without adverse features, the addition of adjuvant therapy did not demonstrate an improvement in OS. This lack of impact was potentially explained by the low frequency of RT or CRT administration in this subgroup, with only 41.5% of patients receiving these treatments, notably lower than expected. Conversely, for individuals with late-stage disease and adverse features, a treatment approach comprising surgery and adjuvant therapy, particularly involving RT, conferred a significant survival advantage over surgery alone. Gordon et al. [7], scrutinizing 12,052 SGC patients, observed a declining trend in the utilization of CRT for both overall patient populations and those with high-risk features. Furthermore, the addition of chemotherapy to RT did not correlate with prolonged survival, as indicated by both propensity score matching and multivariable analyses. Similarly, Aro et al. [5] reported analogous findings, emphasizing that although these studies were based on large NCDB cohorts, discrepancies in defining high-risk variables were noted, potentially resulting in the oversight of critical prognostic factors such as the extent of involvement, PNI, and LVI. In our study, we meticulously accounted for numerous potential adverse characteristics, concluding that CRT did not improved OS, it also conferred minimal impact on the risk of locoregional recurrence compared to RT alone, thus offering a supplementary understanding of SGC management. It is worth noting that certain studies did suggest a longer OS in the CRT group compared to the RT group specifically in cases of salivary gland squamous cell carcinoma [8, 15]. However, caution is advised in interpreting these results, as the potential inclusion of cases with metastasis from cutaneous malignancies could influence these findings.

High-risk variables are widely recognized as significant prognostic indicators for SGC, with the simultaneous presence of multiple adverse features correlating with notably poorer outcomes compared to individual factors. However, the influence of CRT versus RT based on varying numbers of high-risk variables has been scarcely investigated. To the best of our knowledge, only one study has addressed this comparison. In this particular investigation [8], among 6694 patients exhibiting at least one indication for RT, the absence of RT was associated with inferior OS in squamous cell carcinoma and duct carcinoma, with the addition of chemotherapy failing to yield improved survival outcomes beyond those achieved with RT alone. Among 4003 patients presenting with at least one potential indication for CRT, the lack of RT was linked to diminished OS. Furthermore, RT and CRT were found to be associated with comparable OS, albeit CRT employment demonstrated enhanced OS specifically in squamous cell carcinoma cases. This study unveiled that squamous cell carcinoma subtype of SGC predominantly benefited from CRT, positing a potential biological similarity between SGC of squamous cell carcinoma and head and neck squamous cell carcinoma. However, the authors did not delve into the potential outcomes when two or more indications for treatment occurred. We aspire to address this pivotal question, whereby CRT was linked to reduced locoregional recurrence compared to RT when T3/4 stage, high-grade tumor, and presence of 5 or more metastatic lymph nodes were evident. Notably, no notable improvements in LRC or OS were discerned in other subgroups following CRT administration. This intriguing discovery stems from the fact that these three aforementioned factors reflect the actual invasive and metastatic potential of SGC, with the biological behavior potentially modulated by chemotherapy. Nonetheless, the veracity of these findings can only be definitively ascertained upon the publication of results from the forthcoming RTOG 1008 clinical trial.

Limitation in current study must be acknowledge, first, this was a retrospective study, there was inherent selection bias; second, our sample size was not large enough, it might decrease our statistic power, third, further validation is required before clinical application.

In conclusion, the utilization of CRT did not demonstrate a markedly superior outcome in LRC or OS compared to RT in high-risk SGC. Nevertheless, among patients diagnosed with T3/4 stage, high-grade tumors, and 5 or more positive lymph nodes, the inclusion of chemotherapy alongside RT was associated with improved LRC.

### Electronic supplementary material

Below is the link to the electronic supplementary material.

Supplementary Material 1

Supplementary Material 2

## Data Availability

All data generated or analyzed during this study are included in this published article. And the primary data could be achieved from the corresponding author.

## References

[1] Ho AS, Luu M, Balzer BL, Aro K, Jang JK, Mita AC, Scher KS, Mallen-St Clair J, Vasquez M, Bastien AJ, Epstein JB, Lin DC, Chen MM, Zumsteg ZS (2023). Comparative impact of grade on mortality across salivary cancers: a novel, unifying staging system. Head Neck.

[2] Skálová A, Hyrcza MD, Leivo I (2022). Update from the 5th Edition of the World Health Organization Classification of Head and Neck tumors: salivary glands. Head Neck Pathol.

[3] Caudell JJ, Gillison ML, Maghami E, Spencer S, Pfister DG, Adkins D, Birkeland AC, Brizel DM, Busse PM, Cmelak AJ, Colevas AD, Eisele DW, Galloway T, Geiger JL, Haddad RI, Hicks WL, Hitchcock YJ, Jimeno A, Leizman D, Mell LK, Mittal BB, Pinto HA, Rocco JW, Rodriguez CP, Savvides PS, Schwartz D, Shah JP, Sher D, St John M, Weber RS, Weinstein G, Worden F, Yang Bruce J, Yom SS, Zhen W, Burns JL, Darlow SD (2022). NCCN Guidelines® insights: Head and Neck cancers, Version 1.2022. J Natl Compr Canc Netw.

[4] Cheraghlou S, Kuo P, Mehra S, Agogo GO, Bhatia A, Husain ZA, Yarbrough WG, Burtness BA, Judson BL (2018). Adjuvant therapy in major salivary gland cancers: analysis of 8580 patients in the National Cancer Database. Head Neck.

[5] Aro K, Ho AS, Luu M, Kim S, Tighiouart M, Yoshida EJ, Mallen-St Clair J, Shiao SL, Leivo I, Zumsteg ZS (2019). Survival impact of adjuvant therapy in Salivary Gland Cancers following Resection and Neck Dissection. Otolaryngol Head Neck Surg.

[6] Giridhar P, Gupta P, Mallick S, Upadhyay AD, Rath GK (2019). Impact of adjuvant therapy on survival in patients with myoepithelial carcinoma: a systematic review and individual patient data analysis of 691 patients. Radiother Oncol.

[7] Gordon AJ, Chow MS, Patel A, Hu KS, Li Z, Jacobson AS, Vaezi AE, Tam MM, Givi B (2023). Adoption of adjuvant chemotherapy in high-risk salivary gland malignancies. Head Neck.

[8] Patel AM, Haleem A, Choudhry HS, Povolotskiy R, Roden DF (2024). Patterns and trends in Adjuvant Therapy for Major Salivary Gland Cancer. Otolaryngol Head Neck Surg.

[9] Bernier J, Domenge C, Ozsahin M, Matuszewska K, Lefèbvre JL, Greiner RH, Giralt J, Maingon P, Rolland F, Bolla M, Cognetti F, Bourhis J, Kirkpatrick A, van Glabbeke M (2004). European Organization for Research and Treatment of Cancer Trial 22931. Postoperative irradiation with or without concomitant chemotherapy for locally advanced head and neck cancer. N Engl J Med.

[10] Cooper JS, Pajak TF, Forastiere AA, Jacobs J, Campbell BH, Saxman SB, Kish JA, Kim HE, Cmelak AJ, Rotman M, Machtay M, Ensley JF, Chao KS, Schultz CJ, Lee N, Fu KK (2004). Radiation Therapy Oncology Group 9501/Intergroup. Postoperative concurrent radiotherapy and chemotherapy for high-risk squamous-cell carcinoma of the head and neck. N Engl J Med.

[11] Bernier J, Cooper JS, Pajak TF, van Glabbeke M, Bourhis J, Forastiere A, Ozsahin EM, Jacobs JR, Jassem J, Ang KK, Lefèbvre JL (2005). Defining risk levels in locally advanced head and neck cancers: a comparative analysis of concurrent postoperative radiation plus chemotherapy trials of the EORTC (#22931) and RTOG (# 9501). Head Neck.

[12] Amini A, Waxweiler TV, Brower JV, Jones BL, McDermott JD, Raben D, Ghosh D, Bowles DW, Karam SD (2016). Association of Adjuvant Chemoradiotherapy vs Radiotherapy alone with survival in patients with resected major salivary gland carcinoma: data from the National Cancer Data Base. JAMA Otolaryngol Head Neck Surg.

[13] Gebhardt BJ, Ohr JP, Ferris RL, Duvvuri U, Kim S, Johnson JT, Heron DE, Clump DA 2 (2018). Concurrent chemoradiotherapy in the adjuvant treatment of high-risk primary salivary gland malignancies. Am J Clin Oncol.

[14] Kang NW, Kuo YH, Wu HC, Ho CH, Chen YC, Yang CC (2022). No survival benefit from adding chemotherapy to adjuvant radiation in advanced major salivary gland cancer. Sci Rep.

[15] Cheraghlou S, Schettino A, Zogg CK, Otremba MD, Bhatia A, Park HS, Osborn HA, Mehra S, Yarbrough WG, Judson BL (2019). Adjuvant chemotherapy is Associated with Improved Survival for late-stage salivary squamous cell carcinoma. Laryngoscope.

[16] Little R (1988). A test of missing completely at random for multivariable data with missing values. J Am Stat Assoc.

[17] van Buuren S (2007). Multiple imputation of discrete and continuous data by fully conditional specification. Stat Methods Med Res.

[18] Bhushan K, Sharma ML, Gupta DK (2024). Chemotherapy for salivary gland malignant carcinoma: Meta-analysis and systemic review. Indian J Otolaryngol Head Neck Surg.

