# Supplementary material for: Chemoradiotherapy versus radiotherapy in high risk salivary gland cancer
Source: World J Surg Oncol. 2024 Jul 11;22:181. doi: 10.1186/s12957-024-03456-9 (PMC11238513; doi:10.1186/s12957-024-03456-9)
Supplement: Supplementary file 1 — Supplementary Material 1 [file 12957_2024_3456_MOESM1_ESM.doc]

Supplementary Table. Histologic type distribution of salivary gland cancers.

| Cancer type | N |
| --- | --- |
| High grade (n=75) |  |
| Mucoepidermoid carcinoma | 30 |
| Duct carcinoma | 18 |
| Adenocarcinoma not otherwise specified | 11 |
| Spindle cell carcinoma | 9 |
| Large/small cell carcinoma | 7 |
| Intermediate grade (n=102) |  |
| Mucoepidermoid carcinoma | 64 |
| Myoepithelial carcinoma | 30 |
| Adenoid cystic carcinoma | 8 |
| Low grade (n=45) |  |
| Mucoepidermoid carcinoma | 24 |
| Acinic cell carcinoma | 13 |
| Pleomorphic low-grade adenocarcinoma | 3 |
| Basal cell carcinoma | 3 |
| Epithelial-myoepithelial carcinoma | 2 |
