# Supplementary material for: Chemoradiotherapy versus radiotherapy in high risk salivary gland cancer
Source: World J Surg Oncol. 2024 Jul 11;22:181. doi: 10.1186/s12957-024-03456-9 (PMC11238513; doi:10.1186/s12957-024-03456-9)
Supplement: Supplementary file 2 — Supplementary Material 2 [file 12957_2024_3456_MOESM2_ESM.doc]

Table 2. Baseline data of enrolled patients treated by radiotherapy (RT) or chemoradiotherapy (CRT) after propensity score-matching.

| Variable | RT (n=76) | CRT (n=76) | p |
| --- | --- | --- | --- |
| Age |  |  |  |
| ≤50 | 36 (47.4%) | 38 (50.0%) |  |
| >50 | 40 (52.6%) | 38 (50.0%) | 0.746 |
| Sex |  |  |  |
| Male | 30 (39.5%) | 28 (36.8%) |  |
| Female | 46 (60.5%) | 48 (63.2%) | 0.738 |
| Primary site |  |  |  |
| Major | 60 (78.9%) | 64 (84.2%) |  |
| Minor | 16 (21.1%) | 12 (15.8%) | 0.403 |
| Tumor stage |  |  |  |
| T1+T2 | 33 (43.2%) | 35 (46.0%) |  |
| T3+T4 | 43 (56.8%) | 41 (54.0%) | 0.744 |
| Perineural invasion |  |  |  |
| No | 60 (78.9%) | 54 (71.0%) |  |
| Yes | 16 (21.1%) | 22 (29.0%) | 0.261 |
| Lymphovascular invasion |  |  |  |
| No | 61 (80.3%) | 58 (76.3%) |  |
| Yes | 15 (19.7%) | 18 (23.7%) | 0.555 |
| Histologic grade |  |  |  |
| Low | 21 (27.6%) | 12 (15.8%) |  |
| Intermediate | 30 (39.5%) | 35 (46.1%) |  |
| High | 25 (32.9%) | 29 (38.2%) | 0.209 |
| Neck stage |  |  |  |
| N1 | 37 (48.7%) | 37 (48.7%) |  |
| N2/3 | 39 (51.3%) | 39 (51.3%) | 1.000 |
| Level Ⅳ/Ⅴ involvement |  |  |  |
| No | 57 (75.0%) | 54 (71.1%) |  |
| Yes | 19 (25.0%) | 22 (28.9%) | 0.584 |
| Extranodal extension |  |  |  |
| No | 45 (59.2%) | 45 (59.2%) |  |
| Yes | 31 (40.8%) | 31 (40.8%) | 1.000 |
| Margin |  |  |  |
| Negative | 69 (90.8%) | 67 (88.2%) |  |
| Positive | 7 (9.2%) | 9 (11.8%) | 0.597 |
| Number of metastatic lymph nodes |  |  |  |
| ≤4 | 46 (60.5%) | 46 (60.5%) |  |
| >4 | 30 (39.5%) | 30 (39.5%) | 1.000 |
